# A novel approach for characterisation of conformational allergen epitopes combining phage display and high-throughput sequencing

**DOI:** 10.1186/2045-7022-4-S2-P27

**Published:** 2014-03-17

**Authors:** Anders Christiansen, Christian Skjodt Hansen, Jens Vindahl Kringelum, Ole Lund, Katrine Lindholm Bogh, Martin Dufva

**Affiliations:** 1 Technical University of Denmark, Department of Micro- and Nanotechnology, Kgs. Lyngby, Denmark; 2 Technical University of Denmark, Department of Systems Biology, Kgs. Lyngby, Denmark; 3 Technical University of Denmark, National Food Institute, Soborg, Denmark

## Background

Characterization of allergen epitopes is an essential task in unraveling the molecular mechanisms of allergy. At present, the procedures to characterize conformational epitopes are relatively laborious and, consequently, knowledge of conformational allergen epitopes is limited. The aim of this study was to couple an established epitope mapping approach, based on phage-displayed peptide libraries, with high-throughput sequencing in order to make conformational epitope mapping less laborious and increase data output.

## Method

Epitope mimicking sequences were selected from a random 7-mer phage-displayed peptide library over three rounds of biopanning against a monoclonal anti-ovalbumin antibody and competitively eluted by the addition of ovalbumin protein. For all three biopanning rounds, the phage eluate was subjected to PCR and the DNA region containing the displayed peptide was amplified and barcoded. The samples were mixed and sequenced by Ion Torrent Semiconductor Sequencing. The sequences obtained were subject to quality control, aligned (employing a custom-made tool based on the Blast algorithm) and translated into the corresponding peptide sequences. The most abundant peptide sequences were mapped on the three-dimensional structure of ovalbumin using computer-based algorithms.

## Results

The relatively low number of phages obtained after the first biopanning round proved to be sufficient for direct amplification by PCR. As expected in a selection process, the total number of phages increased in subsequent rounds, while the number of unique phages decreased. Tens of thousands of peptide sequences were successfully obtained by high-throughput sequencing. A comparison of the peptide sequences from round 1, 2 and 3 demonstrated the gradual enrichment of certain sequences. The most abundant peptide sequences from round 3 were mapped to the same area on ovalbumin by two separate computer-based algorithms, thus suggesting a potential conformational epitope. At present, this epitope is not characterized in the Immune Epitope Database.

## Conclusion

High-throughput sequencing was successfully coupled with the phage-display technology resulting in a large output of peptide sequence data. The peptide sequences were used to infer the potential localization of a conformational epitope. We anticipate that future studies may exploit the enormous data output to thoroughly characterize the IgE epitope recognition pattern of allergic patients.

